# Comorbidities in unclassifiable interstitial lung disease

**DOI:** 10.1186/s12931-022-01981-3

**Published:** 2022-03-16

**Authors:** Thomas Skovhus Prior, Charlotte Hyldgaard, Sebastiano Emanuele Torrisi, Sissel Kronborg-White, Claudia Ganter, Elisabeth Bendstrup, Michael Kreuter

**Affiliations:** 1 Centre for Rare Lung Diseases, Department of Respiratory Diseases and Allergy, Aarhus University Hospital, Aarhus, Denmark; 2 Diagnostic Center, University Research Clinic for Innovative Patient Pathways, Silkeborg Regional Hospital, Silkeborg, Denmark; 3 Centre for Interstitial and Rare Lung Diseases, Pneumology, Thoraxklinik, University of Heidelberg and German Centre for Lung Research, Heidelberg, Germany

**Keywords:** Unclassifiable interstitial lung disease, Comorbidities, Mortality, Disease course, Cluster analyses

## Abstract

**Background:**

Comorbidities are common in interstitial lung diseases (ILD) and have an important association with survival, but the frequency and prognostic impact of comorbidities in unclassifiable interstitial lung disease (uILD) remains elusive. We aimed to describe the prevalence of comorbidities and assess the impact on survival in patients with uILD. Furthermore, we aimed to identify and characterize potential phenotypes based on clusters of comorbidities and examine their association with disease progression and survival.

**Methods:**

Incident patients diagnosed with uILD were identified at two ILD referral centers in Denmark and Germany from 2003 to 2018. The diagnosis uILD was based on multidisciplinary team meetings. Clinical characteristics and comorbidities were extracted from ILD registries and patient case files. Survival analyses were performed using Cox regression analyses, disease progression was analyzed by linear mixed effects models, and clusters of comorbidities were analyzed using self-organizing maps.

**Results:**

A total of 249 patients with uILD were identified. The cohort was dominated by males (60%), former (49%) or current (15%) smokers, median age was 70 years, mean FVC was 75.9% predicted, and mean DLCO was 49.9% predicted. One-year survival was 89% and three-year survival was 73%. Eighty-five percent of the patients had ≥ 1 comorbidities, 33% had ≥ 3 comorbidities and 9% had ≥ 5 comorbidities. The only comorbidity associated with excess mortality was dyslipidemia. No association between survival and number of comorbidities or the Charlson comorbidity index was observed. Three clusters with different comorbidities profiles and clinical characteristics were identified. A significant annual decline in FVC and DLCO % predicted was observed in cluster 1 and 2, but not in cluster 3. No difference in mortality was observed between the clusters.

**Conclusions:**

The comorbidity burden in uILD is lower than reported in other types of ILD and the impact of comorbidities on mortality needs further clarification. Three clusters with distinct comorbidity profiles were identified and could represent specific phenotypes. No difference in mortality was observed between clusters, but slower disease progression was observed in cluster 3. Better understanding of disease behavior and mortality will require further studies of subgroups of uILD with longer observation time.

## Background

Comorbidities are common in many interstitial lung diseases (ILDs) including idiopathic pulmonary fibrosis (IPF) and hypersensitivity pneumonitis [1–4], but the frequency and prognostic impact in unclassifiable interstitial lung disease (uILD) remains elusive.

In the current classification of ILDs, uILD is acknowledged as a specific disease entity [5]. Cohort studies have previously shown that uILDs are frequently encountered and comprise 10–20% of all ILDs [6–8]. The diagnosis is based on a comprehensive clinical work-up taking all available information into consideration [5, 9]. Management and treatment of this group of patients is challenging, and clinicians have to manage the clinical problems according to the most likely diagnosis. No evidence-based guidelines or treatment recommendations exist, although recent studies described the potential of antifibrotic drugs in patients with progressive uILD [10, 11].

Coexisting comorbidities may further complicate the diagnosis by disallowing cryobiopsies and/or surgical lung biopsies or by simultaneous presentation of overlapping diseases and risk factors such as emphysema and smoking. Furthermore, comorbidities may impact the prognosis and potential treatments, e.g., renal or hepatic disease and ischemic heart disease.

The burden of comorbidities and their association with survival has been characterized in patients with IPF, but there is only limited information about the frequency and type of comorbidities and the impact on survival in patients with uILD [1, 2, 4, 12]. No previous studies have explored combinations of comorbidities and whether they may represent specific phenotypes in uILD. Specific phenotypes may potentially result in different disease courses and prognosis, urge for special attention from health-care providers, cause differences in health-related quality of life (HRQL) or respond differently to treatments.

The aim of the present study was to describe comorbidity patterns in a large cohort of patients with uILD from two expert centers and to assess the impact of the total number and specific type of comorbidities on survival. Furthermore, we aimed to identify and characterize potential phenotypes based on clusters of comorbidities, and to study the association between these clusters and disease progression and survival.

## Methods

We identified incident patients diagnosed with uILD at two ILD referral centers: the Thoraxklinik, Heidelberg University Hospital, Germany and Center for Rare Lung Diseases, Aarhus University Hospital, Denmark during a 16-year period from 2003 to 2018. Clinical characteristics at the time of diagnosis and during follow-up were extracted from the ILD registries at the centers and from patient case files.

We extracted information about age, gender, smoking history including pack years, forced vital capacity (FVC) and forced expiratory volume in 1 s (FEV1) (absolute value and percent predicted), FEV1/FVC ratio and diffusing capacity of the lung for carbon monoxide (DLCO), 6-min walk test distance (6MWD), high-resolution computed tomography (HRCT), bronchoscopy with bronchoalveolar lavage, lung biopsy and comorbidities at the time of diagnosis from patients' medical records or from the ILD registries at the centers [6].

Each case was reviewed by experts and discussed at multidisciplinary team meetings at the treating center based on the available clinical information to ensure that the inclusion criterion *unclassifiable ILD* was met. Patients were considered having unclassifiable ILD when a specific diagnosis could not be reached based on all available clinical information and test results [9].

The clinical characteristics and disease trajectories of patients registered from 2003 to 2009 in Aarhus has been described in a previous publication [6].

Assessment of comorbidities was based on review of the patients’ medical history and medication at baseline. A standardized questionnaire was also included in the assessment of the Heidelberg cohort [13]. Conditions of special interest were pre-specified: emphysema, ischemic heart disease, pulmonary hypertension, diabetes, and gastro-esophageal reflux disease.

## Statistics

Categorical data are presented as frequencies, and continuous data are presented as mean with standard deviation (SD) or median with interquartile range (IQR). To estimate survival, Kaplan–Meier estimates, log-rank test, and univariate and multivariate Cox regression analyses were performed. Adjustments were made for age, gender and FVC in the Cox regression analyses. A linear mixed effects model was used to assess changes in FVC and DLCO in comorbidity clusters during follow-up. Data were analyzed using STATA 14.2 (StataCorp, College Station, Texas).

Clusters of comorbidities were analyzed by computing self-organizing maps (SOMs), also known as Kohonen maps, using Viscovery SOMine 7.2 (Viscovery Software GmbH, Vienna, Austria). Multidimensional data spaces were transformed into lower dimensional abstractions using non-parametric regression analyses. Homogenous data groups were then visualized and analyzed statistically [14] . In a SOM, each color reflects the average frequency of a comorbidity on a fitted color scale. The SOM-Ward Cluster algorithm was applied in the analyses. Data in each cluster were compared to the rest of the cohort (the other clusters combined) using a two-sided t-test with 95% confidence for normally distributed data and the Wilcoxon Mann–Whitney U test otherwise.

## Results

### Patient characteristics

We identified 249 patients with uILD diagnosed between 2003 and 2018; 143 patients were included in Aarhus from 2003–2009 and 2012–2018, and 106 patients in Heidelberg from 2012–2018. Histopathological samples (surgical or cryo lung biopsy) was obtained in 77 (31%) of patients and bronchoalveolar lavage in 173 (69%) of patients. In the remaining cases, no biopsy was performed due to patients’ request, too high risk of biopsy or mild/stable disease. No patients were re-classified to another ILD diagnosis during follow-up. The majority of the patients were males with a smoking history (Table 1). The median follow-up time was 2.0 years; 57 patients (23%) died during follow-up. At baseline, FVC% predicted was mildly reduced whereas DLCO% predicted was moderately reduced. More patients from Aarhus had a smoking history compared to patients from Heidelberg (p = 0.004). In the Aarhus cohort, DLCO%, FVC%, and 6MWD was higher, but only the difference in DLCO% reached statistical significance (p < 0.001, p = 0.1 and p = 0.08, respectively). Baseline characteristics are shown in Table 1.

**Table 1:** Baseline characteristics of the uILD patients

	All patients, n = 249	Aarhus cohort, n = 143	Heidelberg cohort, n = 106
Age, years (IQR)	70.0 (60.0–75.0)	69.0 (60.0–75.0)	70.5 (58.0–77.0)
Male gender, %	60.2	58.7	62.3
Never smokers, %	34.5	27.3	44.3
Current smokers, %	14.9	18.9	9.4
Former smokers, %	49.0	52.4	44.3
Missing, %	1.6	1.4	1.9
Pack years (IQR)	25.0 (10.0–40.0)	30.0 (15.0–40.0)	20.0 (10.0–40.0)
Charlson comorbidity index (IQR)	0 (0–1)	0 (0–1)	0 (0–1)
FVC, % pred (SD)	75.9 (24.6)	78.1 (24.9)	73.0 (23.9)
DLCO, % pred (SD)	49.9 (20.3)	54.1 (20.8)	44.6 (18.3)
6MWD, m (SD)	357.4 (136.2)	372.3 (142.5)	338.1 (125.6)
Follow up, years (IQR)	2.0 (0.8–3.3)	1.8 (0.9–2.9)	2.5 (0.7–3.6)
1-year survival (95% CI)	0.89 (0.84–0.92)	0.88 (0.81–0.92)	0.91 (0.82–0.95)
3-year survival (95% CI)	0.73 (0.66–0.79)	0.67 (0.56–0.76)	0.81 (0.70–0.88)

Data are presented as frequencies, mean with standard deviation (SD), median with interquartile range (IQR) or survival with 95% confidence intervals (CI). *FVC*: Forced vital capacity, *DLCO*: diffusion capacity of the lung for carbon monoxide, *6MWD*: distance walked during the 6-min walk test

### Number of comorbidities

**Fig. 1:**
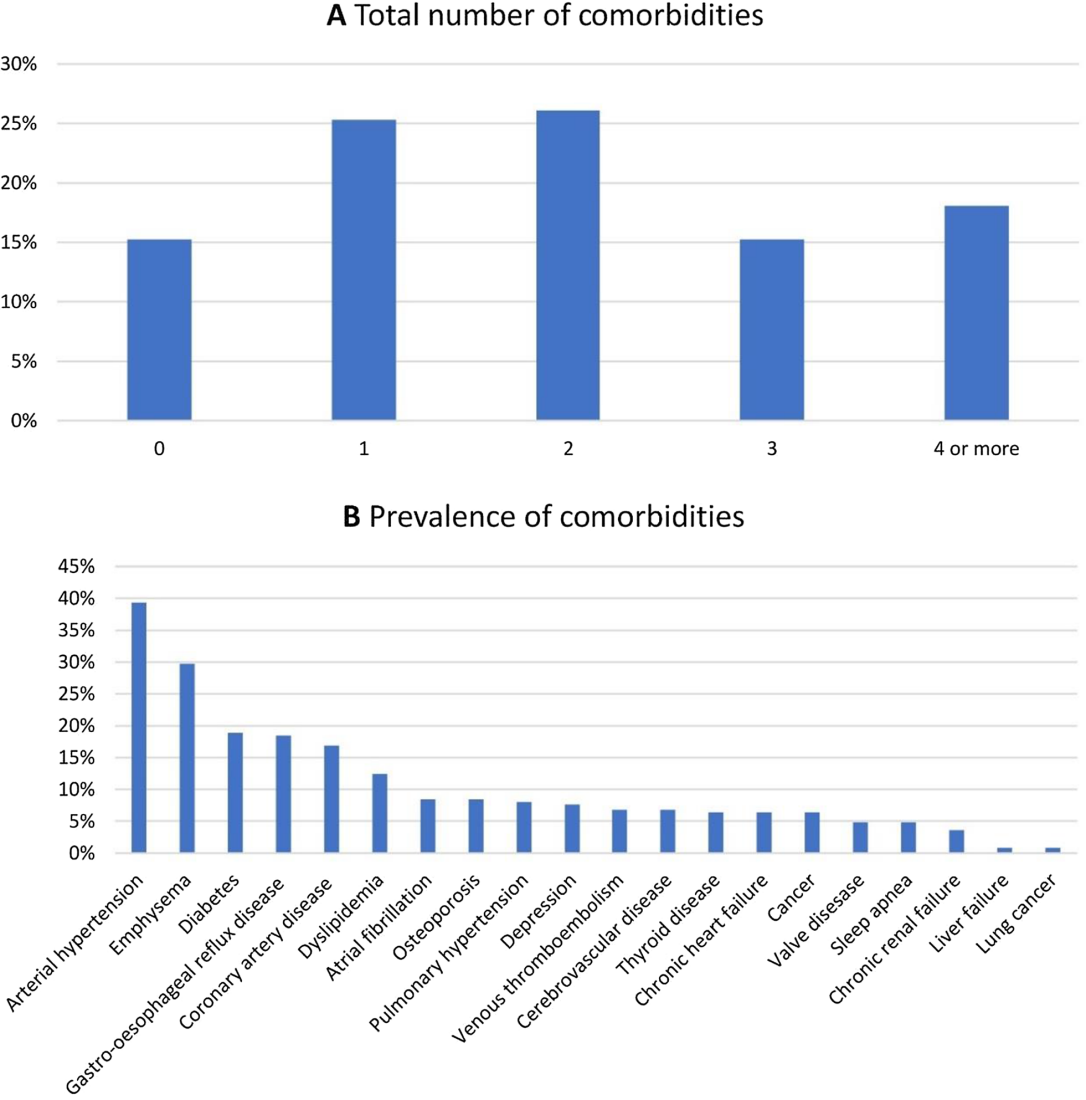
Total and specific comorbidities in the cohort. Data are presented as a percentage of all patients. **A** Total number of comorbidities per patient. **B** Spectrum of comorbidities in the uILD cohort. Multiple comorbidities could be reported

Eighty-five percent of the patients had one or more comorbidities (Fig. 1a). The median number of comorbidities was two (IQR 1–3), and the median Charlson comorbidity index was zero (IQR 0–1). The frequency of the registered comorbidities ranged from 1 to 39%. Arterial hypertension (39%), emphysema (30%), diabetes (19%), gastro-esophageal reflux (18%) and coronary artery disease (17%) were most common. Some slight differences between centers were seen in specific comorbidities, potentially due to different smoking prevalences. The baseline prevalence of all comorbidities is presented in Fig. 1b.

### Survival analysis

The only comorbidity associated with excess mortality was dyslipidemia in both univariate and multivariate analyses (Table 2). None of the five pre-specified comorbidities of special interest (emphysema, ischemic heart disease, pulmonary hypertension, diabetes, and gastro-esophageal reflux disease) were associated with excess mortality. No survival difference was seen in patients with zero comorbidities vs. 1–3 or zero vs. 4 or more comorbidities, nor in analyses of patients stratified by the median number of comorbidities or median Charlson comorbidity index (Table 3).

**Table 2:** Survival analyses for specific comorbidities

Comorbidities	Univariate analysis (95% CI)	Multivariate analysis (95% CI)
Diabetes (n = 47)	1.20 (0.63–2.29)	0.93 (0.48–1.83)
Pulmonary hypertension (n = 20)	1.66 (0.75–3.68)	1.52 (0.67–3.43)
Lung cancer (n = 2)	3.19 (0.44–23.40)	2.32 (0.31–17.32)
Emphysema (n = 74)	1.04 (0.56–1.91)	1.24 (0.65–2.37)
Gastro-esophageal reflux disease (n = 46)	0.92 (0.46–1.83)	0.95 (0.46–1.96)
Arterial hypertension (n = 98)	1.25 (0.74–2.11)	1.02 (0.58–1.79)
Depression (n = 19)	1.75 (0.79–3.86)	1.75 (0.74–4.13)
Valve disease (n = 12)	1.18 (0.37–3.80)	0.85 (0.26–2.77)
Atrial fibrillation (n = 21)	1.66 (0.75–3.69)	0.90 (0.38–2.16)
Dyslipidemia (n = 31)	2.16 (1.18–3.96)	2.25 (1.19–4.24)
Coronary artery disease (n = 42)	1.35 (0.71–2.56)	1.06 (0.53–2.10)
Cancer (non-lung) (n = 16)	1.75 (0.69–4.40)	1.16 (0.44–3.06)
Osteoporosis (n = 21)	1.14 (0.49–2.65)	1.20 (0.50–2.87)
Cerebrovascular disease (n = 17)	1.85 (0.79–4.32)	1.64 (0.68–3.97)
Venous thromboembolism (n = 17)	0.80 (0.25–2.58)	0.48 (0.12–2.01)
Thyroid disease (n = 16)	0.64 (0.20–2.05)	0.74 (0.17–3.20)
Sleep apnea (n = 12)	*	*
Chronic heart failure (n = 9)	1.97 (0.84–4.61)	1.41 (0.58–3.42)
Chronic renal failure (n = 9)	0.83 (0.20–3.41)	0.58 (0.14–2.42)
Liver failure (n = 2)	*	*

Data are presented as hazard ratios with 95% confidence intervals (CI). Hazard ratios > 1 indicate an association with increased mortality. Multivariate analyses are adjusted for gender, age, and FVC% predicted. *: Number of deaths too low for analysis

**Table 3:** Survival analyses for number of comorbidities and Charlson comorbidity index

Parameter	Univariate analysis (95% CI)	Multivariate analysis (95% CI)	1-year survival (95% CI)	3-year survival (95% CI)
*Number of comorbidities*				
0	Ref	Ref	0.91 (0.74–0.97)	0.80 (0.56–0.92)
1–3	1.07 (0.47–2.41)	0.78 (0.34–1.81)	0.90 (0.83–0.94)	0.74 (0.65–0.82)
4 or more	1.40 (0.56–3.50)	0.90 (0.35–2.31)	0.86 (0.71–0.93)	0.64 (0.46–0.78)
≤ median (0–2)	Ref	Ref	0.89 (0.83–0.93)	0.79 (0.70–0.86)
> median (3 or more)	1.46 (0.86–2.49)	1.12 (0.64–1.95)	0.88 (0.78–0.94)	0.63 (0.49–0.74)
*Charlson comorbidity index*				
≤ median (0)	Ref	Ref	0.88 (0.79–0.93)	0.77 (0.64–0.85)
> median (1 or more)	1.17 (0.68–2.03)	0.75 (0.42–1.35)	0.90 (0.83–0.94)	0.71 (0.61–0.79)

Data are presented as hazard ratios or percent survivors with 95% confidence intervals (CI). Hazard ratios > 1 indicate an association with increased mortality. Multivariate analyses are adjusted for gender, age, and FVC% predicted. *Ref.*: Reference group

### Clusters of comorbidities

**Fig. 2:**
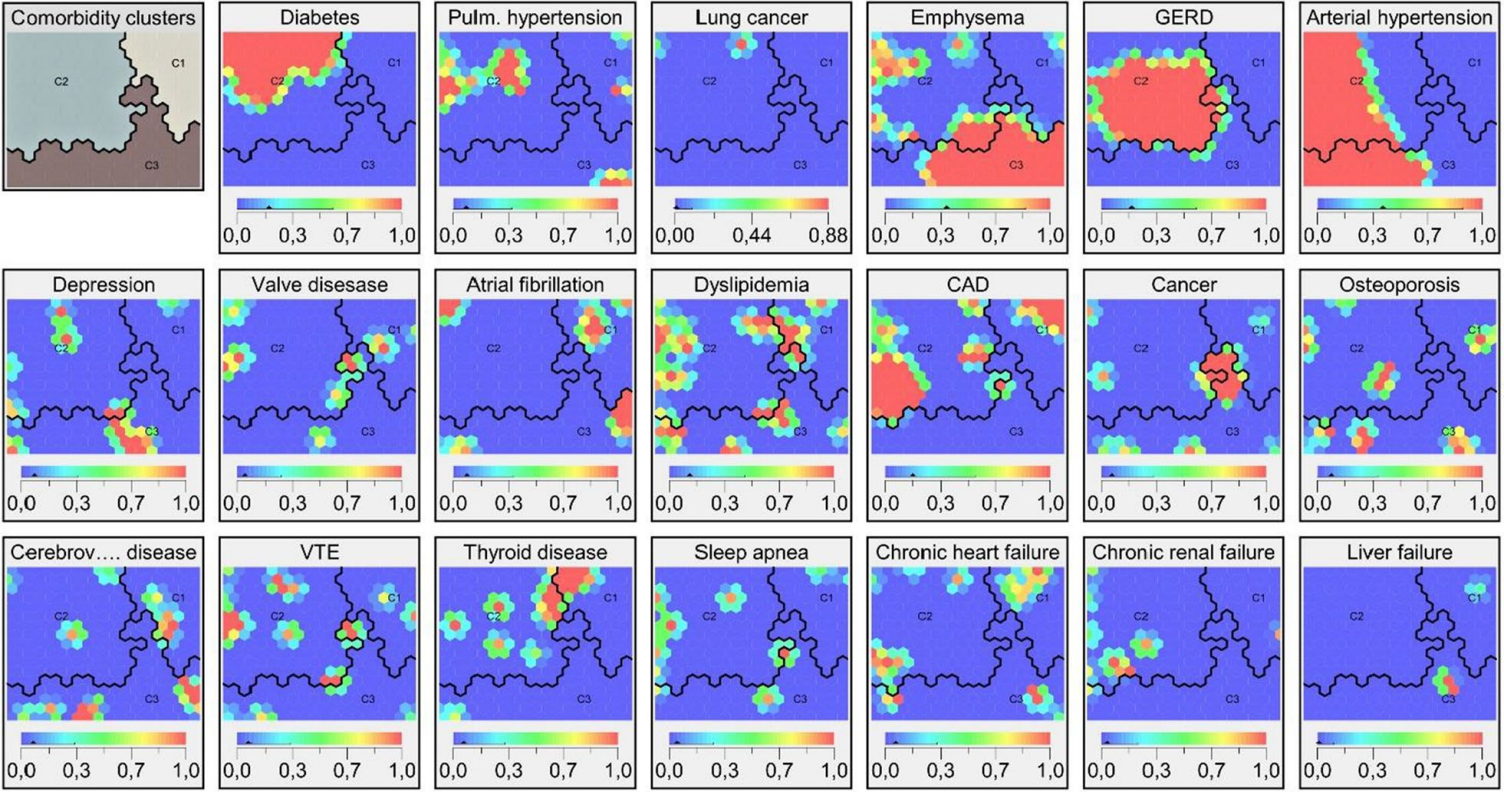
Comorbidity clusters and heat maps of each comorbidity. Cluster borders are indicated by the black lines. Each patient is placed in the same area on all maps. Red colors indicate a high frequency of the specific comorbidity, while blue colors indicate absence of the comorbidity. *C1*: Cluster 1; *C2*: Cluster 2; *C3*: Cluster 3; *GERD*: Gastro-esophageal reflux disease; *CAD*: Coronary artery disease; *VTE*: Venous thromboembolism

**Fig. 3:**
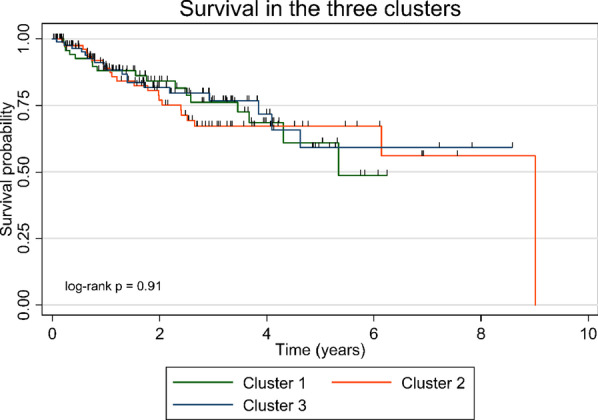
Survival in the three clusters

Three clusters with different comorbidity profiles were identified (Fig. 2, Table 4). Patients in cluster 1 had significantly fewer comorbidities than the entire cohort. Cluster 2 was dominated by patients with a larger total number of comorbidities, primarily cardiovascular and associated diseases, a higher body mass index (BMI) and more severely impaired pulmonary function and exercise capacity based on six-minute walk test Emphysema, cancer and depression were more prevalent in cluster 3, and these patients had a lower BMI and better exercise capacity. No difference in mortality was observed between the clusters (Fig. 3, Table 4). A significant annual decline in FVC and DLCO % predicted was observed in cluster 1 and 2, but not in cluster 3 (Table 5).

**Table 4:** Clinical characteristics and prevalence of comorbidities in the three comorbidity clusters

	Cluster 1: (Few comorbidities) n = 77	Cluster 2: (Cardiovascular) n = 85	Cluster 3: (Emphysema) n = 87
Age, years (IQR)	68.0 (57.0–75.0), p = 0.22	69.0 (58.0–75.0), p = 0.99	71.0 (61.0–77.0), p = 0.23
Male, %	59.7, p = 0.91	63.5, p = 0.45	57.5, p = 0.51
Never smokers, %	36.4, p = 0.78	37.8, p = 0.53	31.4, p = 0.37
Current smokers, %	13.0, p = 0.53	12.2, p = 0.37	19.8, p = 0.13
Former smokers, %	50.6, p = 0.86	50.0, p = 0.96	48.8, p = 0.83
Missing, %	0.0	3.5	1.2
Pack years (IQR)	20.0 (15.0–30.0), p = 0.18	25.5 (13.0–40.0), p = 0.47	35.0 (10.0–43.0), p = 0.56
Body mass index (SD)	27.2 (5.3), p = 0.14	29.8 (4.9), p = 0.001^†^	26.8 (4.8), p = 0.03*
FVC, % predicted (SD)	77.4 (21.3), p = 0.52	71.1 (26.4), p = 0.03*	79.3 (24.8), p = 0.11
DLCO, % predicted (SD)	53.8 (20.5), p = 0.06	44.5 (19.7), p = 0.006*	51.6 (19.8), p = 0.36
6MWD, m (SD)	377.7 (123.6), p = 0.19	304.2 (139.5), p < 0.0001*	394.6 (127.2), p = 0.005^†^
Total number of comorbidities, n (SD)	0.9 (1.1), p < 0.0001*	3.4 (2.0), p < 0.0001^†^	2.0 (1.1), p = 0.41
Emphysema, %	5.7, p < 0.0001*	25.7, p = 0.01*	70.3, p < 0.0001^†^
Pulmonary hypertension, %	3.9, p = 0.11	14.1, p = 0.01^†^	5.7, p = 0.33
Venous thromboembolism, %	3.9, p = 0.22	11.8, p = 0.03^†^	4.6, p = 0.31
Sleep apnea, %	2.6, p = 0.28	8.2, p = 0.07	3.4, p = 0.46
Coronary artery disease, %	22.1, p = 0.14	28.2, p = 0.0005^†^	1.1, p < 0.0001*
Dyslipidemia, %	6.5, p = 0.06	20.0, p = 0.009^†^	10.3, p = 0.46
Arterial hypertension, %	0.0, p < 0.0001*	63.5, p < 0.0001^†^	50.6, p = 0.008^†^
Atrial fibrillation, %	10.4, p = 0.46	7.1, p = 0.58	8.0, p = 0.87
Chronic heart failure, %	7.8, p = 0.56	8.2, p = 0.40	3.4, p = 0.16
Heart valve disease, %	3.9, p = 0.65	8.2, p = 0.07	2.3, p = 0.17
Cerebrovascular disease, %	7.8, p = 0.69	3.5, p = 0.14	9.2, p = 0.28
Chronic renal failure, %	1.3, p = 0.19	8.2, p = 0.005^†^	1.1, p = 0.13
Diabetes, %	0.0, p < 0.0001*	55.3, p < 0.0001^†^	0.0, p < 0.0001*
Osteoporosis, %	5.2, p = 0.22	8.2, p = 0.94	11.5, p = 0.20
Gastro-esophageal reflux disease, %	0.0, p < 0.0001*	51.8, p < 0.0001^†^	2.3, p < 0.0001*
Thyroid disease, %	7.8, p = 0.56	9.4, p = 0.17	2.3, p = 0.05
Liver failure, %	1.3, p = 0.56	0.0, p = 0.31	1.1, p = 0.66
Lung cancer, %	0.0, p = 0.34	2.4, p = 0.049^†^	0.0, p = 0.30
Cancer, %	1.3, p = 0.03*	4.7, p = 0.43	12.6, p = 0.003†
Depression, %	1.3, p = 0.01*	7.1, p = 0.81	13.8, p = 0.007†

Data are presented as means with standard deviations (SD) or interquartile range (IQR) for continuous variables and frequencies for categorical variables. Significance levels for the comorbidities was based on comparison between the result in one cluster and the rest of the cohort (the two other clusters combined) using the t-test or Wilcoxon Mann–Whitney U test

*Significantly lower or less frequent in this cluster compared with the rest of the cohort (the two other clusters combined). ^†^Significantly higher or more frequent in this cluster compared with the rest of the cohort (the two other clusters combined). *FVC*: Forced vital capacity, *DLCO*: diffusion capacity of the lung for carbon monoxide, *6MWD*: distance walked during the 6-min walk test

**Table 5:** Survival analyses and changes in pulmonary function during follow-up

Parameter	Cluster 1 n = 77	Cluster 2 n = 85	Cluster 3 n = 87
*Survival analyses*			
Number of deaths (%)	17 (22%)	22 (26%)	18 (21%)
Univariate analysis (95% CI)	Ref	1.07 (0.56 to 2.03)	0.93 (0.48 to 1.81)
Multivariate analysis (95% CI)	Ref	0.82 (0.42 to 1.61)	0.84 (0.42 to 1.67)
*Pulmonary function*			
ΔFVC, % predicted	− 2.59 (− 3.44 to − 1.74)	− 2.17 (− 3.06 to − 1.27)	− 0.54 (− 1.54 to 0.47)
ΔDLCO, % predicted	− 1.62 (− 2.60 to − 0.64)	− 1.37 (− 2.36 to − 0.37)	− 0.95 (− 2.05 to 0.16)

Data are presented as frequencies, hazard ratios (Cox regression analyses), or change pr. Year (linear mixed effects models) with 95% confidence intervals. Multivariate analyses are adjusted for gender, age, and FVC% predicted. Δ: Change per year. *FVC*: Forced vital capacity, *DLCO*: diffusion capacity of the lung for carbon monoxide. *Ref.*: Reference group

Patients were followed for a median time of 2 years

## Discussion

Unclassifiable ILDs are associated with a severe prognosis compared to most other ILDs, approaching the prognosis of IPF [9]. However, beside the characteristics describing progressive pulmonary fibrosis in this cohort, other factors contributing to mortality are not well understood. As patients with uILD are mainly elderly, comorbidities could have an important impact on outcomes. The present study shows that comorbidities are common in patients with uILD, but the burden and impact of comorbidities were less pronounced in uILD than in other fibrotic ILDs [1–4]. We found a median number of two comorbidities, whereas Prior et al. reported a median of six comorbidities in IPF [4] and Wälscher et al., reported a median number of three in chronic hypersensitivity pneumonitis (cHP) [3]. These differences were observed even though our uILD patients were older (median age 70 years) than patients with cHP (mean age 63 years), or the same age as patients with IPF (67.4 to 72.9 years). A larger proportion of our patients (34.5%) were never smokers compared to IPF (19.0–26.7%), which may partly explain the difference in the comorbidity burden [4, 6]. Krauss et al. reported 38.6% never smokers in their uILD cohort, which was similar to our findings in the present study, but lower than in cHP (50.2%) [3, 12]. The spectrum of comorbidities included and differences in the approach to registration may also influence the total number of comorbidities. Overall, patients with uILD seem to have fever comorbidities compared with other ILDs. Our data support the most recent ILD classification characterizing uILD as a specific diagnostic entity [5].

The most prevalent comorbidities were the same as reported in other comorbidity studies in fibrotic ILD. Two studies have compared the prevalence of comorbidities in patients with ILD with age- and gender matched controls in the general population and showed a higher comorbidity burden in ILD in general [15, 16]. Arterial hypertension is consistently reported as the most common comorbidity in fibrotic ILD [1–4, 12, 17]. Gastro-esophageal reflux disease (GERD) was among the five most common comorbidities, similar to what was observed in IPF and uILD [1, 2, 4, 12, 17]. The influence of GERD on disease progression in IPF has been repeatedly discussed and data on anti-acid treatment and surgical fundoplication are conflicting [18, 19].

Similarly, emphysema and coronary artery disease were common in our uILD cohort and percentages varied only little compared to other uILD, IPF and cHP cohorts, despite a large number of never-smokers. It still remains to be seen whether smoking and environmental and genetic risk factors or the underlying ILD disease is the most significant contributor to emphysema. Diabetes is a frequent comorbidity in IPF and other ILDs and was observed in almost 20% of our cohort [3, 4, 12, 20]. The relationship between diabetes and fibrotic and/or inflammatory ILD has previously been described, but it is unclear whether diabetes is a potential risk factor for disease development or disease progression [21].

In IPF and cHP, several studies have shown an association between mortality and the burden of comorbidities, expressed either as numbers, frequencies, or using the Charlson comorbidity index [2, 3, 22]. This was not the case in our study. The only comorbidity associated with increased mortality was dyslipidemia, whereas related diseases such as coronary artery disease, chronic heart failure, chronic renal disease and cerebrovascular disease showed no clear association with mortality. These dissimilarities may be caused by lack of power due to the limited number of patients included in the present study or differences in age, gender and smoking characteristics compared to IPF and cHP, or they may be related to other factors such as physical (in-) activity, diet, social background, and educational level [23]. Unfortunately, these factors cannot be characterized based on the available data in this study. Furthermore, the effect on survival might be mediated by the use of statins, as these have antioxidant and anti-inflammatory properties [24, 25], and may limit fibrosis by inhibiting lung fibroblasts [26, 27]. The survival observed in our cohort of patients with uILD was better compared with IPF. Due to the better prognosis, longer follow-up may be needed to see the impact of comorbidities in uILD.

Three distinct clusters were identified based on overall similarities in comorbidity patterns and revealed interesting associations between comorbidities and clinical parameters, possibly representing phenotypes in uILD. Cluster 1 consisted of patients with fewer comorbidities than the rest of the cohort, whereas patients in cluster 2 had the largest number of comorbidities per patient, mainly cardiovascular and associated diseases. Their lower pulmonary function and exercise capacity could be due to more advanced uILD and the higher prevalence of pulmonary hypertension, which could be caused by cardiac disease. Cluster 3 had a high prevalence of emphysema. Pulmonary hypertension was not frequent in this cluster, and we did not observe the association between combined pulmonary fibrosis and emphysema and PH that has been described in other studies [28]. Patients in cluster 1 experienced an annual decline in FVC% predicted, as did patients in cluster 2. The insignificant decline in FVC% predicted in cluster 3 may be explained by the high prevalence of emphysema, as uILD and emphysema have inverse impacts on FVC. No difference in mortality was observed between the clusters. It would probably require longer follow-up due to the low number of patients in each cluster and the relatively low mortality. Similar comorbidity clusters have been observed in patients with IPF [4]. Four cluster were identified, and the comorbidity profiles of the first three cluster were comparable to our findings in uILD: a cluster of patients with few comorbidities, a second cluster of patients with more comorbidities dominated by cardiovascular diseases, a third cluster predominantly consisting of patients with emphysema, and a fourth cluster of patients with a high prevalence of anxiety and depression and more comorbidities than the rest of the cohort. Likewise, a significant decline in pulmonary function was observed in the fewer comorbidities and cardiovascular clusters, and the IPF study also showed similar survival across the four clusters. Contrary to our results, there was an association between smoking history and number of comorbidities in IPF. The similarities between the clusters in IPF and uILD support the robustness of this stratification. Future studies should investigate the clinical implications of comorbidity clusters to further characterize these potential phenotypes.

Our study and other studies of uILD have shown that this entity is much more heterogeneous than IPF. A study by Hyldgaard et al. showed that disease behavior was able to predict mortality [9], but further studies on disease behavior in subgroups of uILD are needed. The increasing knowledge of non-IPF ILD overall, and especially the subgroup of patients with progressive fibrosing ILDs, will also contribute to knowledge of uILD, which is especially needed for patients with severe and progressive uILD.

### Strengths and limitations

The strengths of our study are the large cohort of patients and the inclusion of data from two expert centers. Both centers have implemented a standardized collection of comorbidity data, which increases the probability of identifying the most important comorbidities. Our study has a number of limitations. As the study is retrospective, there is always an inherent risk of bias due to incomplete data collection and may therefore not be as generalizable as a prospective study aimed at registering all comorbidities. We did not register co-medication and cannot account for their impact on other comorbidities or long-term clinical outcomes. Also, disease-specific treatments such as corticosteroid treatment may have affected the prevalence and severity of comorbidities such as osteoporosis and diabetes. The limited follow-up time and sample size could influence the mortality analyses. A larger study with longer follow-up time would be able to further elucidate the association between comorbidities and mortality.

## Conclusion

The comorbidity burden in uILD is lower than reported in other types of ILD and the impact of comorbidities on mortality needs further clarification. Three clusters with distinct comorbidity profiles were identified and could represent specific phenotypes. No difference in mortality was observed between clusters, but slower disease progression was observed in patients in cluster 3. Better understanding of disease behavior and mortality will require further studies of subgroups of uILD with longer observation time.

## Data Availability

The datasets generated and/or analyzed during the current study are not publicly available due to restrictions by the Danish and German data protection laws but are available from the corresponding author on reasonable request.

## Abbreviations

6MWD: 6-Minute walk test distance
BMI: Body mass index
cHP: Chronic hypersensitivity pneumonitis
CI: Confidence interval
DLCO: Diffusing capacity of the lung for carbon monoxide
FEV1: Forced expiratory volume in 1 s
FVC: Forced vital capacity
GERD: Gastro-esophageal reflux disease
HRCT: High-resolution computed tomography
HRQL: Health-related quality of life
ILD: Interstitial lung diseases
IPF: Idiopathic pulmonary fibrosis
IQR: Interquartile range
SD: Standard deviation
SOM: Self-organizing maps
uILD: Unclassifiable interstitial lung disease

## References

[1] Hyldgaard C, Hilberg O, Bendstrup E (2014). How does comorbidity influence survival in idiopathic pulmonary fibrosis?. Respir Med.

[2] Kreuter M, Ehlers-Tenenbaum S, Palmowski K, Bruhwyler J, Oltmanns U, Muley T (2016). Impact of comorbidities on mortality in patients with idiopathic pulmonary fibrosis. PLoS ONE.

[3] Wälscher J, Gross B, Morisset J, Johannson KA, Vasakova M, Bruhwyler J (2020). Comorbidities and survival in patients with chronic hypersensitivity pneumonitis. Respir Res..

[4] Prior TS, Hoyer N, Hilberg O, Shaker SB, Davidsen JR, Rasmussen F (2021). Clusters of comorbidities in idiopathic pulmonary fibrosis. Respir Med..

[5] Travis WD, Costabel U, Hansell DM, King TE, Lynch DA, Nicholson AG (2013). An Official American Thoracic Society/European Respiratory Society Statement: update of the international multidisciplinary classification of the idiopathic interstitial pneumonias. Am J Respir Crit Care Med.

[6] Hyldgaard C, Hilberg O, Muller A, Bendstrup E (2014). A cohort study of interstitial lung diseases in central Denmark. Respir Med..

[7] Thomeer MJ, Vansteenkiste J, Verbeken EK, Demedts M (2004). Interstitial lung diseases: characteristics at diagnosis and mortality risk assessment. Respir Med.

[8] Ryerson CJ, Urbania TH, Richeldi L, Mooney JJ, Lee JS, Jones KD (2013). Prevalence and prognosis of unclassifiable interstitial lung disease. Eur Respir J.

[9] Hyldgaard C, Bendstrup E, Wells AU, Hilberg O (2017). Unclassifiable interstitial lung diseases: clinical characteristics and survival. Respirology.

[10] Maher TM, Corte TJ, Fischer A, Kreuter M, Lederer DJ, Molina-Molina M (2020). Pirfenidone in patients with unclassifiable progressive fibrosing interstitial lung disease: a double-blind, randomised, placebo-controlled, phase 2 trial. Lancet Respir Med.

[11] Flaherty KR, Wells AU, Cottin V, Devaraj A, Walsh SLF, Inoue Y (2019). Nintedanib in progressive fibrosing interstitial lung diseases. N Engl J Med..

[12] Krauss E, El-Guelai M, Pons-Kuehnemann J, Dartsch RC, Tello S, Korfei M (2020). Clinical and functional characteristics of patients with unclassifiable interstitial lung disease (uILD): long-term follow-up data from European IPF Registry (eurIPFreg). J Clin Med..

[13] Kreuter M, Ochmann U, Koschel D, Behr J, Bonella F, Claussen M (2018). DGP interstitial lung disease patient questionnaire. Pneumologie.

[14] Kohonen T. Self-Organizing Maps [Internet]. Berlin, Heidelberg: Springer Berlin Heidelberg; 2001. 10.1007/978-3-642-56927-2

[15] Hilberg O, Bendstrup E, Løkke A, Ibsen R, Fløe A, Hyldgaard C (2018). Co-morbidity and mortality among patients with interstitial lung diseases: a population-based study. Respirology.

[16] Collard HR, Ward AJ, Lanes S, Cortney Hayflinger D, Rosenberg DM, Hunsche E (2012). Burden of illness in idiopathic pulmonary fibrosis. J Med Econ.

[17] Raghu G, Amatto VC, Behr J, Stowasser S (2015). Comorbidities in idiopathic pulmonary fibrosis patients: a systematic literature review. Eur Respir J.

[18] Lee JS, Collard HR, Anstrom KJ, Martinez FJ, Noth I, Roberts RS (2013). Anti-acid treatment and disease progression in idiopathic pulmonary fibrosis: an analysis of data from three randomised controlled trials. Lancet Respir Med.

[19] Kreuter M, Wuyts W, Renzoni E, Koschel D, Maher TM, Kolb M (2016). Antacid therapy and disease outcomes in idiopathic pulmonary fibrosis: a pooled analysis. Lancet Respir Med.

[20] Kim YJ, Park JW, Kyung SY, Lee SP, Chung MP, Kim YH (2012). Clinical characteristics of idiopathic pulmonary fibrosis patients with diabetes mellitus: the national survey in Korea from 2003 to 2007. J Korean Med Sci.

[21] Kopf S, Groener JB, Kender Z, Fleming T, Brune M, Riedinger C (2018). Breathlessness and restrictive lung disease: an important diabetes-related feature in patients with type 2 diabetes. Respiration.

[22] Glaspole I, Bonella F, Bargagli E, Glassberg MK, Caro F, Stansen W (2021). Efficacy and safety of nintedanib in patients with idiopathic pulmonary fibrosis who are elderly or have comorbidities. Respir Res..

[23] Montano D (2021). Socioeconomic status, well-being and mortality: a comprehensive life course analysis of panel data, Germany, 1984–2016. Arch Public Health.

[24] Shishehbor MH, Brennan ML, Aviles RJ, Fu X, Penn MS, Sprecher DL (2003). Statins promote potent systemic antioxidant effects through specific inflammatory pathways. Circulation.

[25] Jain MK, Ridker PM (2005). Anti-inflammatory effects of statins: clinical evidence and basic mechanisms. Nat Rev Drug Discov..

[26] Watts KL, Sampson EM, Schultz GS, Spiteri MA (2005). Simvastatin inhibits growth factor expression and modulates profibrogenic markers in lung fibroblasts. Am J Respir Cell Mol Biol..

[27] Oka H, Ishii H, Iwata A, Kushima H, Toba S, Hashinaga K (2013). Inhibitory effects of pitavastatin on fibrogenic mediator production by human lung fibroblasts. Life Sci.

[28] Bolaki M, Antoniou KM (2020). Combined pulmonary fibrosis and emphysema. Semin Respir Crit Care Med..

